# A Newly Established Cuproptosis-Related Gene Signature for Predicting Prognosis and Immune Infiltration in Uveal Melanoma

**DOI:** 10.3390/ijms241411358

**Published:** 2023-07-12

**Authors:** Wei Huang, Fan Yang, Yichi Zhang, Qianqi Fang, Yitao Lai, Yuqing Lan

**Affiliations:** 1 Department of Ophthalmology, Sun Yat-Sen Memorial Hospital, Sun Yat-Sen University, Guangzhou 510120, China; huangw298@mail.sysu.edu.cn (W.H.);; 2 Guangdong Provincial Key Laboratory of Malignant Tumor Epigenetics and Gene Regulation, Sun Yat-Sen Memorial Hospital, Sun Yat-Sen University, Guangzhou 510120, China; 3 Center for Reproductive Genetics and Reproductive Medicine, Sun Yat-Sen Memorial Hospital, Sun Yat-Sen University, Guangzhou 510120, China

**Keywords:** UVM, cuproptosis, gene signature, prognostic value, immune microenvironment

## Abstract

Uveal melanoma (UVM) is the most common primary ocular malignancy in adults and involves several types of regulated cell death. Cuproptosis is a novel method of regulating cell death by binding lipoylated TCA cycle proteins. There is still no research on the relationship between cuproptosis-related genes (CRGs) and UVM. Here, we aimed to develop a prognostic CRG signature for UVM. After a prognostic CRG signature was constructed, we determined the relationship between the signature and immune infiltration, bioinformatics analysis and experimental validation. Finally, a prognostic cuproptosis-related three-gene (CRTG) signature was constructed, which comprised ORAI2, ACADSB and SLC47A1. The risk score of the CRTG signature was negatively correlated with the overall survival (OS) and progression-free survival (PFS) of patients, which revealed strong predictive ability and its independent prognostic value. In addition, we found that the risk score was negative for chromosomes 3 and 6p, and positive for 8q, and high-risk UVM patients showed an increase in protumor immune infiltrates and a high expression of immune checkpoints. Finally, experimental validation verified that the migratory ability of MUM-2B cells was suppressed by the knockdown of the identified genes in vitro. We constructed a CRTG signature that is helpful in predicting prognosis and guiding treatment for patients with UVM.

## 1. Introduction

Uveal melanoma (UVM) is the most common primary ocular malignancy in adults, although still rare, with an incidence varying from one to nine per million people per year [1] UVM is a subtype of melanoma that is different from cutaneous and other types of melanoma in terms of its biological and clinical characteristics, and it mostly arises from the choroid (90%), iris (6%), and ciliary body (4%) [2]. The 5-year and 15-year disease-related mortality rates of UVM are ~30% and ~45% [3]. Almost half of all UVM patients will develop metastatic disease (usually via hematogenous spread), resulting in an extremely high mortality rate [4].

The symptoms of UVM depend on the location of the tumor, including blurring, distorted vision, and loss of the visual field. Moreover, ~30% of patients are asymptomatic and the tumor is discovered incidentally during routine ocular examination [5]. Primary disease treatment aims to conserve the eye and improve vision, usually combined with local resection, radiotherapy, immunotherapy, chemotherapy, and phototherapy. However, there are currently no effective therapies for preventing metastasis [6]. Unlike cutaneous melanoma metastases, UVM metastases generally respond poorly to immune checkpoint inhibitors and chemotherapy [6]. In addition, given the substantial mortality and lack of prospective diagnostic tools, particularly novel predictive models and useful biomarkers, there is an urgent need to develop more efficient prognostic models.

Various types of cell death are closely associated with the formation, progression, survival, and metastasis of cancer, including ferroptosis, necroptosis, autophagy, and pyroptosis [7]. Recently, copper (Cu) was reported to be involved in a novel means of cell death named “cuproptosis”, which was first reported by Tsvetkov et al. [8]. Cu is an essential cofactor for all organisms; however, excess Cu is toxic. Excess copper directly binds to the lapidated components of the tricarboxylic acid cycle, leading to lipid-acylated protein aggregation and subsequent iron–sulfur cluster protein loss, which results in proteotoxic stress, and ultimately, cell death [8]. Subsequently, several studies have mined the prognostic gene signatures related to cuproptosis in tumors. Lv demonstrated the prognostic value of cuproptosis-related genes, especially lipoyltransferase 1 (LIPT1), and revealed a correlation between LIPT1 expression and immune infiltration in melanoma [9]. Wang et al. revealed a novel cuproptosis-based signature to predict the prognosis, biological features, and appropriate treatment of patients with glioma [10]. Zhang et al. created a prognostic lncRNA profile linked to cuproptosis to predict the response to immunotherapy in hepatocellular carcinoma [11]. 

However, there is no research on whether a cuproptosis-related gene (CRG) signature can predict the prognosis of UVM. In this study, we constructed a cuproptosis-related three-gene (CRTG) signature utilizing the Cancer Genome Atlas (TCGA) UVM dataset (TCGA-UVM) and the GSE22138 dataset from the Gene Expression Omnibus (GEO), along with CRGs identified in previous studies [8]. The signature revealed an association between the cuproptosis-related genes and the tumor microenvironment, providing new insights into the role of cuproptosis in UVM. In addition, the CRTG signature could be used to identify patients who are likely to have higher sensitivity to immune checkpoint therapies.

## 2. Results

**Figure 1:**
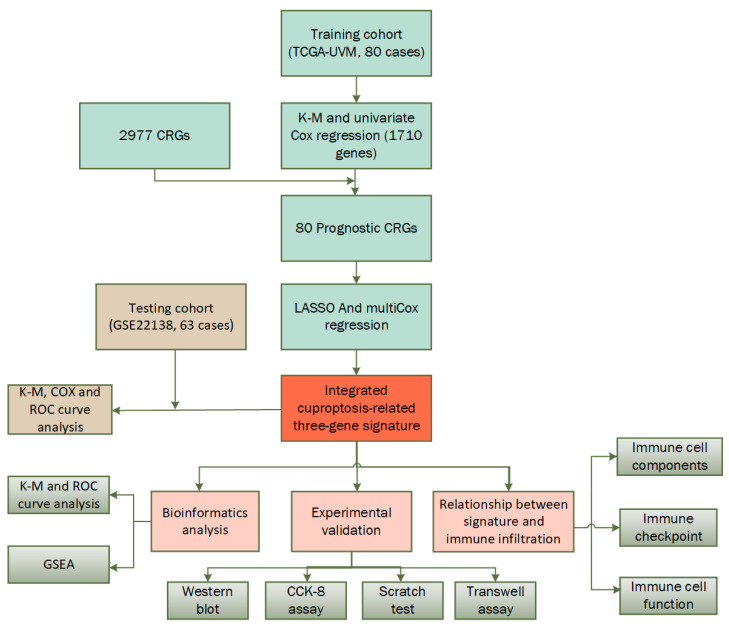
Flow chart of the study.

In Figure 1, a flow chart of the present study is shown, while the clinical characteristics of all the UVM patients in this study are summarized in Table 1. There was no significant difference in clinical characteristics between the training cohort and testing cohort. In this study, the training cohort included 80 cases from the TCGA-UVM dataset and the testing cohort included 63 cases from the GSE22138 dataset.

### 2.1. Construction of a Prognostic CRTG Signature

**Figure 2:**
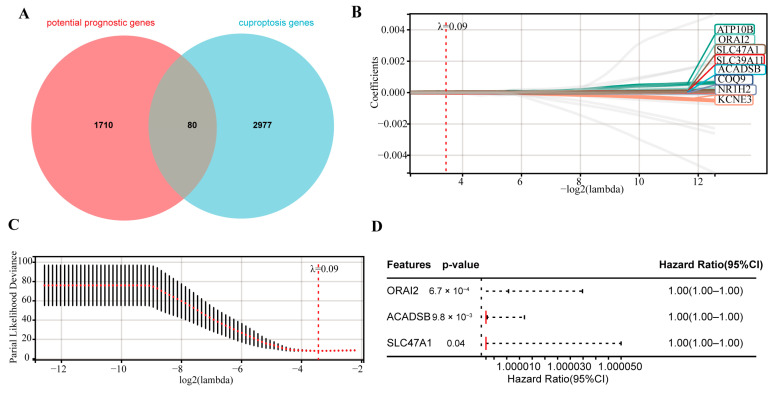
Construction of the CRTG signature using LASSO and multivariate Cox regression. (**A**) Venn diagram indicating 80 prognostic CRGs. (**B**) Distribution of the LASSO coefficients of the 8 prognostic CRGs in the training cohort. (**C**) The generated coefficient distribution plots for the logarithmic (lambda) sequence for the selection of the best parameter (lambda). (**D**) Forest plot of the three CRGs screened via multivariate Cox regression.

In the training cohort, 1710 potential prognostic genes were identified using Kaplan–Meier (K–M) and univariate Cox analyses (*p* < 0.05). Next, 80 prognostic CRGs were identified as overlapping genes of the 1710 potential prognostic genes and 2977 CRGs (Figure 2A). These 80 potential prognostic CRGs (Table S1) were constructed into an overall survival (OS)-based least absolute shrinkage and selection operator (LASSO) Cox regression model, and eight prognostic CRGs were screened (Figure 2B,C). Finally, based on the OS, a subsequent multivariate Cox analysis highlighted three CRGs for prognosis: calcium release-activated calcium modulator 2 (ORAI2), acyl-CoA dehydrogenase short/branched chain (ACADSB), and solute carrier family 47 member 1 (SLC47A1) (Figure 2D).

### 2.2. Verifying the Prognostic Capacity of the CRTG Signature

**Figure 3:**
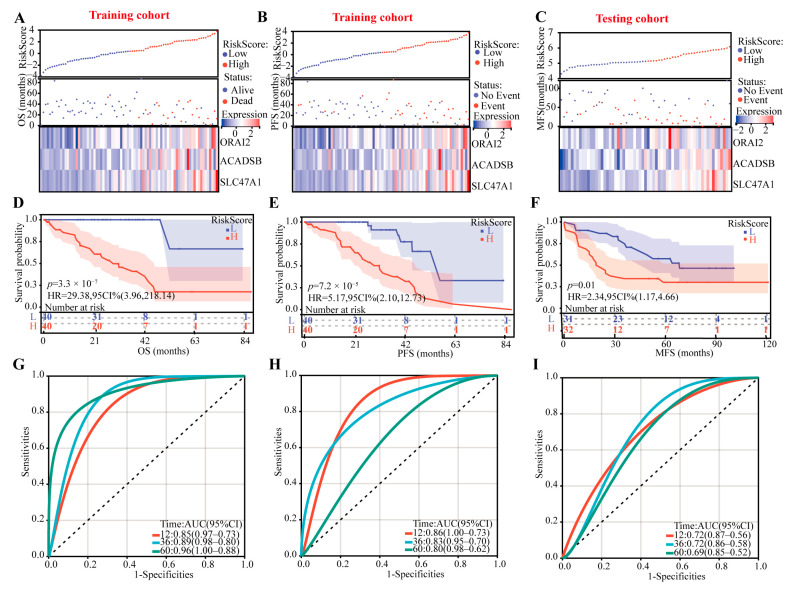
Identifying the prognostic ability of the CRTG signature in both the training and testing cohorts. (**A**–**C**) The distributions of the risk scores and patients’ survival times and the expression heatmaps of ORAI2, ACADSB, and SLC47A1. (**D**–**F**) Kaplan–Meier curves of the CRTG signature risk score. (**G**–**I**) ROC curves of the CRTG signature.

In this prognostic CRTG signature model, the risk score = 1.6560 × 10^−6^ × ACADSB + 6.7730 × 10^−6^ × ORAI2 + 1.8441 × 10^−5^ × SLC47A1, and using the median risk score as a cutoff point, the patients were divided into low-risk and high-risk groups. All the patients’ risk scores, survival outcomes, and three-gene expression levels are shown in Figure 3A–C, and we found that the patients with lower risk had better survival outcomes in both the training and testing cohorts. In addition, the K–M curves showed that the high-risk patients had poor OS and progression-free survival (PFS) in the training cohort and metastasis-free survival in the testing cohort (*p* < 0.01 all, Figure 3D–F).

According to the time-dependent receiver operating characteristic (ROC) analysis, the CRTG signature has great predictive ability, with areas under curve of 0.85, 0.89, and 0.96 (based on OS) and 0.86, 0.83, and 0.80 (based on PFS) in the training cohort and 0.72, 0.72, and 0.69 in the testing cohort at one, three, and five years, respectively (Figure 3G–I).

**Figure 4:**
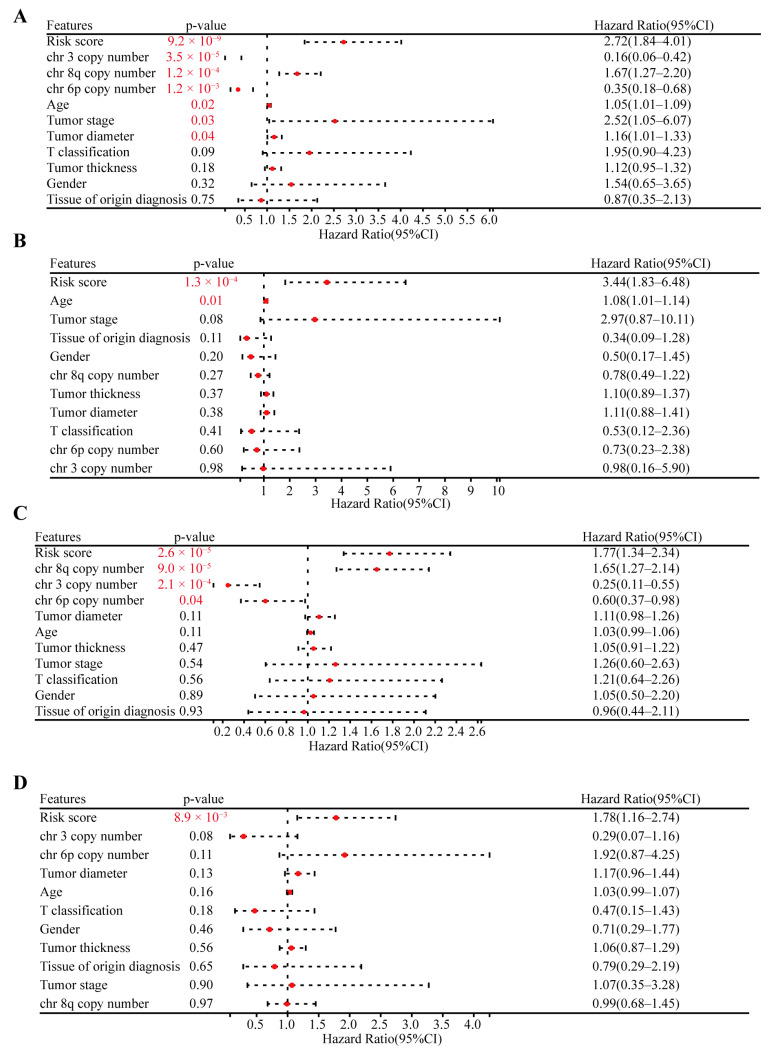
Validating the independent prognostic capacity of the CRTG signature with other clinical characteristics in the training cohort. (**A**,**B**) Univariate analysis and multivariate analysis of the correlation of the CRTG signature risk score with other clinical characteristics based on the OS of the UVM patients. (**C**,**D**) Univariate analysis and multivariate analysis of the correlation of the CRTG signature risk score with other clinical characteristics based on the PFS of the UVM patients. The red *p*-value indicates statistical significance.

Finally, based on the OS and PFS data, we used both univariate and multivariate Cox regression to validate the prognostic capacity and independence of the CRTG signature with other clinical characteristics, including age, sex, tumor stage, tumor T classification, tissue of origin diagnosis, tumor thickness, tumor diameter, and the copy number of chromosomes 3/6p/8q. As shown in Figure 4A,B, the risk score of the CRTG signature and age were the risk factors for OS (risk score: hazard ratio (HR) = 2.72, *p* = 9.2 × 10^−9^; HR = 3.44, *p* = 1.3 × 10^−4^). In addition, in Figure 4C,D, the risk score was the only risk factor for PFS in the TCGA-UVM patients (HR = 1.77, *p* = 2.6 × 10^−5^; HR = 1.78, *p* = 8.9 × 10^−3^). In conclusion, the CRTG signature was an independent prognostic variable.

### 2.3. Correlation Analysis of the CRTG Signature and UVM Common Mutations

**Figure 5:**
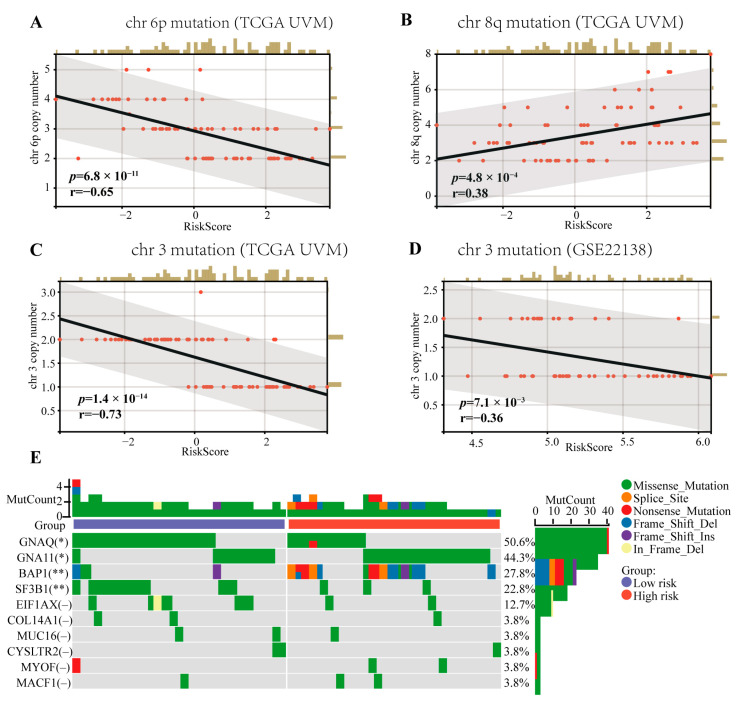
Correlations between the CRTG signature risk score and the chromosome aberrations of UVM and the mutation landscape of the CRTG signature. (**A**,**B**) The risk score of the CRTG signature was negative with the chr 6p copy number and positive with the chr 8q copy number in the training cohort. (**C**,**D**) The risk score of the CRTG signature was negative with the chr 3 copy number in both the training and testing cohorts. (**E**) Mutation landscape of the CRTG signature: the top 10 most frequently mutated genes in all the patients from the TVGA-UVM dataset. The black line in each graph fits a linear model that indicates the proportional trend of the chromosome aberrations and the risk score. The grey shading around the black line indicates the 95% confidence interval. The brown columns and red dots represent UM cases. The en dash indicates no statistical significance. * *p* < 0.05 and ** *p* < 0.01.

Mutations in chromosomes 3/6p/8q, which are risk factors for patient prognosis, are common in UVM patients. As shown in Figure 5, we verified that the copy number of chromosomes 3/6p/8q was related to the patients’ OS and PFS via univariate Cox regression (HR = 0.16, *p* = 3.5 × 10^−5^; HR = 0.35, *p* = 1.2 × 10^−3^; HR = 1.67, *p* = 1.2 × 10^−4^; HR = 0.25, *p* = 2.1 × 10^−4^; HR = 0.60, *p* = 0.04; HR = 1.65, *p* = 9.0 × 10^−5^, respectively). Furthermore, the CRTG signature was negatively correlated with the chromosome 3 copy number in both the training and testing cohorts (R = −0.69, *p* = 9.3 × 10^−13^; R = −0.37, *p* = 5.8 × 10^−3^, respectively, Figure 5C,D), negatively correlated with the chromosome 6p copy number (R = −0.62, *p* = 1.0 × 10^−9^, Figure 5A), and positively correlated with the chromosome 8q copy number (R = 0.41, *p* = 1.3 × 10^−4^, Figure 5B) in the training cohort. The top 10 most frequently mutated genes in all the patients from the TVGA-UVM dataset are shown in Figure 5E Among these 10 genes, GNAQ and SF3B1 mutations were significantly more frequent in the low-risk group (*p* < 0.05, *p* < 0.01), while GNA11 and BAP1 mutations were significantly more frequent in the high-risk group (*p* < 0.05, *p* < 0.01).

### 2.4. Functional Enrichment Analysis

**Figure 6:**
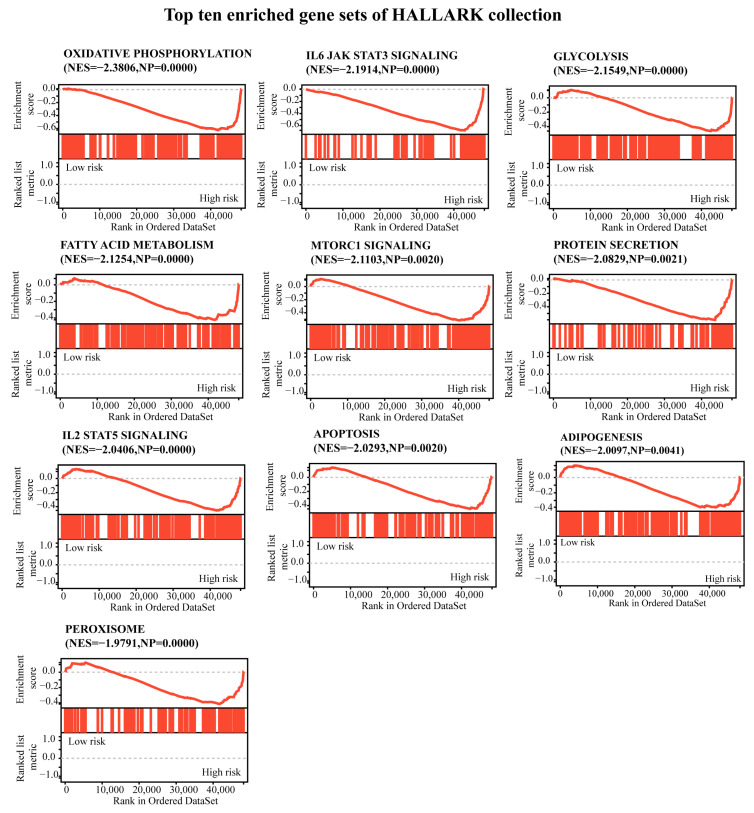
A gene set enrichment analysis was performed using the HALLMARK collection. |NES| > 1, NOM *p* < 0.05, and FDR q < 0.25 were set as the significance thresholds.

A gene set enrichment analysis (GSEA) was conducted between the low-risk and high-risk groups, as shown in Figure 6 and Table S2, and the enriched gene sets in the high-risk group were mainly involved in mechanisms related to oxidative phosphorylation, IL-6 jak stat3 signaling, glycolysis, fatty acid metabolism, mtorc1 signaling, protein secretion, IL-2 stat5 signaling, apoptosis, adipogenesis, and peroxisome.

### 2.5. Correlation Analysis of the CRTG Signature and Immune Cell Infiltration

**Figure 7:**
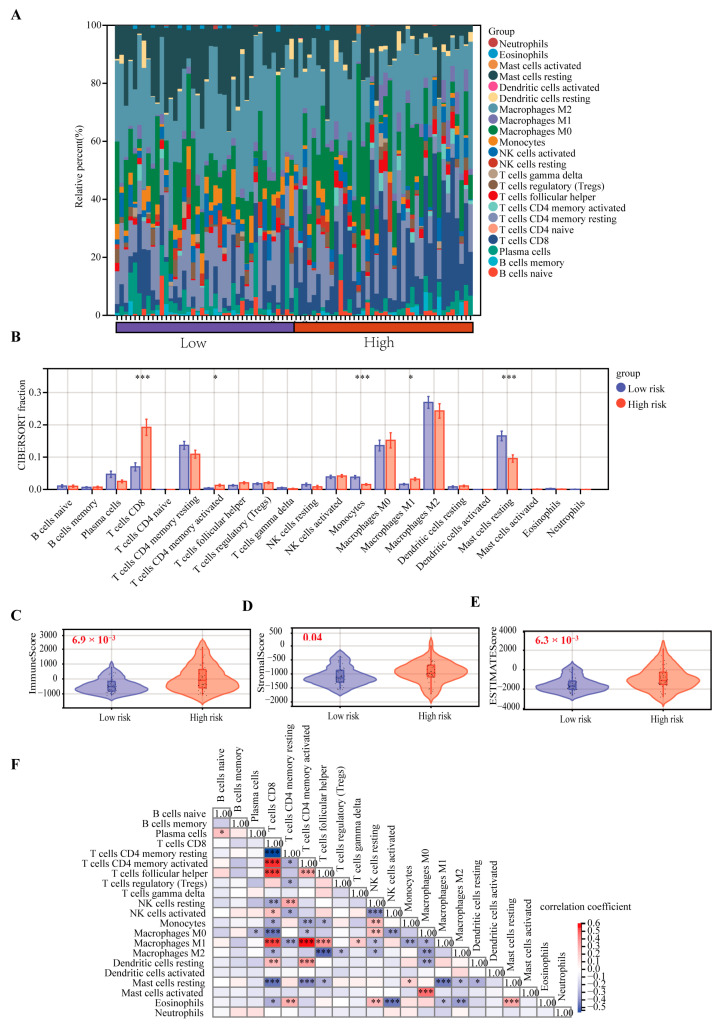
Comparison of the 22 TICs between the low-risk and high-risk groups. (**A**) Heatmap of the 22 TICs in all the patients. (**B**) Comparing the proportions of the 22 TICs between the low-risk and high-risk groups. (**C**–**E**) Comparison of the immune, stromal, and estimate scores. (**F**) Heatmap of the correlation of the immune cells. * *p* < 0.05, ** *p* < 0.01, and *** *p* < 0.001.

The CIBERSORT algorithm was used to identify the relationship between the CRTG signature and tumor immune microenvironment in the UVM patients. As shown in Figure 7A, a heatmap showed the changes in the 22 tumor-infiltrating immune cells (TICs) between the low- and high-risk groups. A comparison of the differences in the 22 TICs was made between the two groups (Figure 7B), and we found that the percentages of CD8 T cells, CD4 memory activated and macrophages M1 were significantly upregulated, although the monocytes and resting mast cells were significantly downregulated in the high-risk group. Furthermore, the immune, stromal, and estimate scores were significantly higher in the high-risk group than in the low-risk group (*p* = 6.9 × 10^−3^, 0.04, 6.3 × 10^−3^, respectively; Figure 7C–E). The correlation between the different immune cells is shown in Figure 7F.

**Figure 8:**
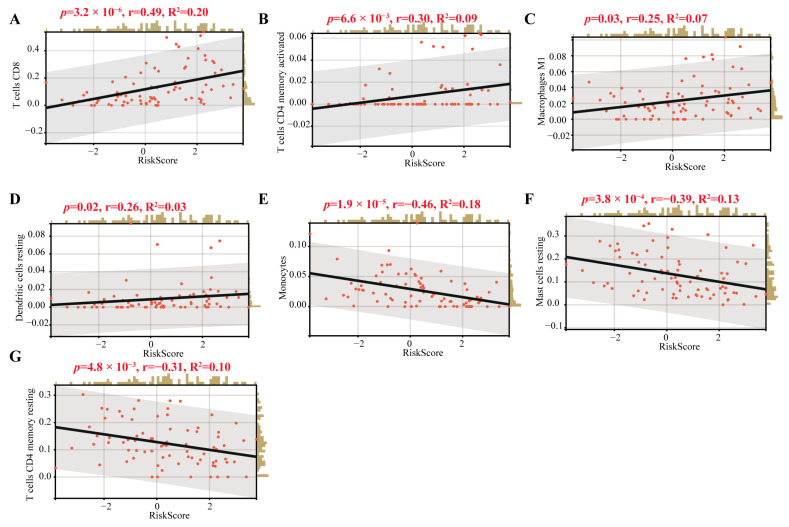
Correlations of the TICs and the CRTG signature. (**A**–**G**): The black line in each graph fits a linear model that indicates the proportional trend of the TICs and the risk score. The grey shading around the black line indicates the 95% confidence interval. The brown columns and red dots represent UM cases.

**Figure 9:**
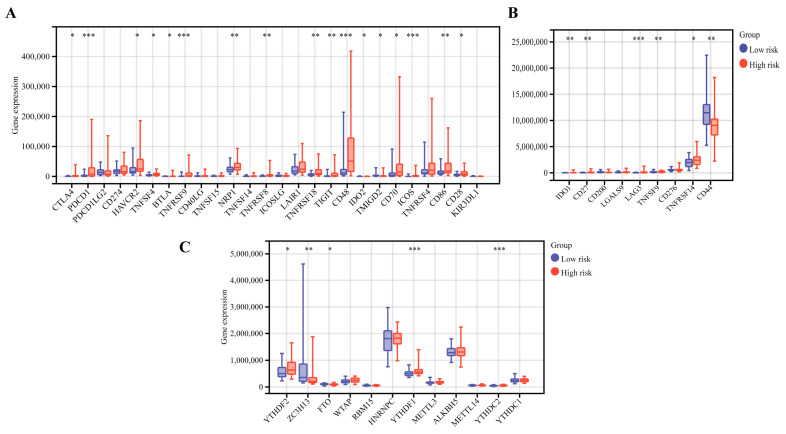
The differential gene expression of the immunotherapy checkpoints (**A**,**B**) and m6A (**C**) between the low-risk and high-risk groups. * *p* < 0.05, ** *p* < 0.01, and *** *p* < 0.001.

Interestingly, we found that T cells CD8, CD4 memory activated, macrophages M1 and dendritic resting were positively correlated with the CRTG signature, whereas monocytes, mast cells resting and T cells CD4 memory resting were negatively correlated with it (Figure 8). As shown in Figure 9A,B, 23 validated effective checkpoint immunotherapy targets were overexpressed in the high-risk group, including CTLA4, PDCD1, HAVCR2, TNFSF4, BTLA, and TNFRSF9, and six N6-methyladenosine (m6A)-related genes were overexpressed in the high-risk group, including YTHDF2, ZC3HI3, FTO, YTHDF1, and YTHDC2. These results revealed that the immune responses of the two groups were different and so provided a beneficial basis for antitumor immunotherapy for UVM.

### 2.6. Experimental Validation Analysis

The effect of cuproptosis on the viability of MUM-2B cells was tested by means of a cell viability assay. The cell viability decreased in the cells treated with elesclomol-CuCl_2_; however, the inhibitive effect could be reversed by treatment with cuproptosis inhibitor TTM (Supplementary Figure S1), indicating the role of cuproptosis in the cell viability of UVM.

**Figure 10:**
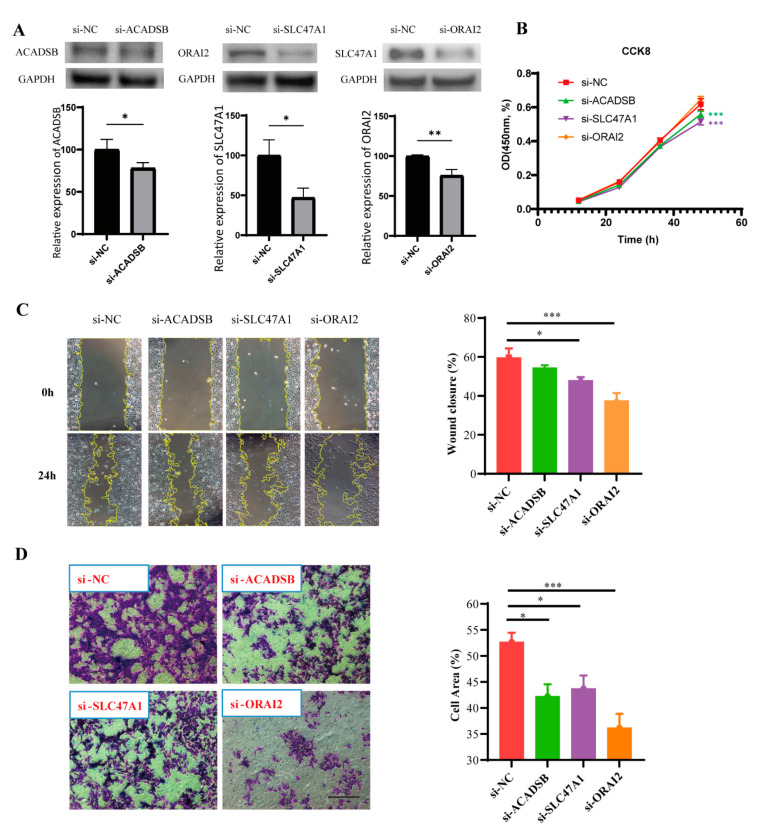
The biological effect of the CRTG signature on MUM-2B cells. (**A**) The transfection efficiency was verified via Western blot. (**B**–**D**) After the knockdown of three identified CRTGs, the cell proliferation and migration abilities of MUM-2B cells were assessed via CCK-8 assay, scratch test and transwell assay. Scale bar = 250 μm (**C**,**D**). * *p* < 0.05, ** *p* < 0.01, and *** *p* < 0.001.

To verify the prognostic function of the CRTG signature further, we used the MUM-2B cell line. The transfection efficiency was confirmed via Western blotting (Figure 10A), as the expressions of ACADSB, SLC47A1, and ORAI2 were dramatically reduced by siRNA. We found that the downregulation of ACADSB and SLC47A1 significantly inhibited the proliferation of MUM-2B cells, although the downregulation of ORAI2 had no significant inhibitory effect (Figure 10B). Furthermore, the scratch test showed that the scratches healed slowly after SLC47A1 and ORAI2 knockdown, suggesting that SLC47A1 and ORAI2 knockdown reduced the migration ability of MUM-2B cells (Figure 10C). Moreover, the migration ability was assessed via transwell assay, and the results showed that knockdown of ACADSB, SLC47A1, and ORAI2 reduced the migration ability of MUM-2B cells (Figure 10D). In conclusion, these findings indicate that ACADSB, SLC47A1, and ORAI2 are risk factors in MUM-2B cells and that their high expression promotes UVM cancer growth in some way.

## 3. Discussion

An increasing number of new forms of regulated cell death have been identified in ocular diseases, such as necroptosis [12] and PANoptosis [13]. As the newest type of regulated cell death, cuproptosis is caused by copper binding directly to the lipoylated components of the tricarboxylic acid cycle, leading to toxic protein stress. An increasing number of studies have shown a correlation between cuproptosis and the outcomes of some cancers. However, we are the first to explore the prognostic function of CRGs in UVM.

In the present study, we discovered a CRTG signature that showed strong prognostic prediction capabilities in both the training and testing cohorts. Our signature comprised three CRGs: ORAI2, ACADSB, and SLC47A1. In this signature model, all three CRGs were unfavorable for the prognosis of UVM. The Orai channel family is a highly Ca^2+^-selective channel family that consists of three homologs: ORAI1, ORAI2, and ORAI3. ORAI2 is widely expressed, and its biophysical profile is similar to that of ORAI1, which appears to be the most likely candidate for mediating the remaining store-operated Ca^2+^ currents and Ca^2+^ signals [14]. The significance of ORAI2-mediated Ca^2+^ signaling in cancer hallmarks remains poorly understood. Recently, Wu et al. found that a high frequency of ORAI2-positive cells in gastric cancer tissues was significantly correlated with poor differentiation, invasion, lymph node metastasis, and poor prognosis, which was demonstrated in both gastric cancer cell lines and mice [15]. Sanchez-Collado et al. demonstrated that ORAI2 plays a functional role in agonist-evoked Ca^2+^ signaling, cell proliferation, and apoptosis resistance in breast cancer cell lines [16]. Kumar-Singh et al. also found that ORAI1 and ORAI2 regulate oral cancer cell migration and colonization [17]. However, our study is the first to report a correlation between ORAI2 and the UVM outcome.

Acyl-coenzyme A dehydrogenase, short/branched chain (ACADSB, also named SBCAD) is a member of the acyl-CoA dehydrogenase family, which usually exists in the mitochondria and catalyzes the dehydrogenation of acyl-CoA derivatives [18]. Lu et al. uncovered that the expression of ACADSB was positively correlated with the OS rate of colorectal cancer but negatively correlated with lymph node metastasis and distant metastasis [19]. Liu et al. also found that low ACADSB expression was an independent risk factor for OS in clear cell renal carcinoma patients, and they revealed that ACADSB was downregulated in multiple cancers and negatively correlated with poor prognosis in certain types of cancer using a pan-cancer analysis [20]. However, in our study, we found that ACADSB expression was positively correlated with the OS and PFS in UVM patients.

SLC47A1 encodes multidrug and toxin extrusion transporter-1 (MATE1), which is a key membrane transporter. Yao et al. discovered that MATE1 (SLC47A1) could be the single most predictive marker of chemosensitivity to platinum–acridines and demonstrated its potential as a target for personalized cancer treatment [21].

Previous studies have confirmed that certain chromosomes are aberrations and that genes are mutated in UVM patients, which are very closely related to the prognosis, including chromosomes 3, 8q, and 6p [22]. The monosomy of chromosome 3 is associated with a high metastatic risk for UVM patients, and increased chromosome 8q and lack of 6p gain are associated with poor prognosis [23]. In our study, the risk score of the CRTG signature was negative for chromosomes 3 and 6p and positive for 8q (Figure 5), which is consistent with previous studies. This result supports the importance of our CRTG signature in predicting the UVM prognosis.

UVM patients with a high-risk CRTG signature might have more resistance to cuproptosis than patients with a low-risk signature. Cells undergoing glycolysis or hypoxia are less sensitive to cuproptosis [8]. In our study, the GSEA analysis showed that the high-risk UVM patients were enriched in the glycolysis, oxidative phosphorylation, and reactive oxygen species pathways (Figure 6, Table S2). It is likely that UVM patients with a high risk might have an aggressive phenotype and show a poor prognosis, probably because they have acquired the ability to attenuate copper-induced toxicity.

Next, we found that our CRTG signature was closely related to the tumor immune microenvironment in patients with UVM. Both the CIBERSORT algorithm and Spearman coefficient showed significantly higher levels of CD8^+^ T cells, CD4 T cells and macrophages M1 infiltration in the high-risk group, which was positively correlated with the risk of the CRTG signature. Thus, UVM patients with a high-risk score may be associated with inflamed phenotypes. Through a single-cell analysis of metastasized UVM, Durante et al. also found that the CD8 T cells and the exhausted subtypes were the main infiltrated immune cells [24]. These findings suggest that the low response rates of UVM to the immune checkpoint therapy may result from its low tumor mutation burden and relatively immune-excluded tumor environment [25].

Immune checkpoint inhibitors can suppress the tumor immune microenvironment by reducing the immune cells [26,27]. Among them, anti-programmed death-1 (PD-1) and anti-CTLA-4 have already been used to treat patients with metastatic UVM in the clinic [28,29], and a meta-analysis showed that immune checkpoint blockade immunotherapy was helpful in improving the OS of UVM patients [30]. Recent studies have shown that m6A RNA modification plays an essential role in both physiological and pathological conditions, especially in the initiation and progression of different types of human cancers. The m6A RNA modification process offers potential targets for cancer therapy in the future [31]. In this study, we found that some checkpoint immunotherapy targets were overexpressed in the high-risk group, including PDCD1 and CTLA4, and that the levels of CD8+ T cell infiltration were significantly higher. Wang et al. revealed that CD8+ T cells could regulate the endocytic recycling of PD1 and exert a synergistic effect with anti-PD1 therapy in hepatocellular carcinoma [32]. These findings highlight a promising strategy for enhancing UVM immunotherapy.

In summary, we created and confirmed a novel CRTG signature to predict the prognosis of UVM, which could be used as a potential biomarker and for the development of effective immunotherapy for UVM patients.

## 4. Materials and Methods

### 4.1. Cohorts and CRGs

RNA sequencing data in the FPKM format and the clinicopathological characteristics of UVM (*n* = 80) were downloaded from the TCGA (http://portal.gdc.cancer.gov/, accessed on 31 July 2022), which was chosen as the training cohort. For the testing cohort, dataset GSE22138 and the clinicopathological data of UVM (*n* = 63) were downloaded from the GEO (http://www.ncbi.nlm.nih.gov/geo/, accessed on 31 July 2022). In addition, CRGs were identified from previous literature [8].

### 4.2. Identification and Validation of the Prognostic Cuproptosis-Related Gene Signature

Potential prognostic genes were screened using K–M and univariate Cox regression in the training cohort, which showed significance in both analyses. Potential cuproptosis prognostic genes were identified as overlapping genes of the potential prognostic genes and CRGs. The prognostic cuproptosis-related gene signature was constructed by combining LASSO Cox regression and multivariate Cox regression analyses in the training cohort. A LASSO Cox regression analysis with five cross-validations was conducted by applying the “glmnet” R package. Finally, the risk score for each individual was calculated using the prognostic signature, where *n*, *Expri*, and *βi* represent the number of hub genes, gene expression level, and regression coefficient value, respectively.

(1) $$\mathrm{Risk}\;\mathrm{score}=\sum_{i}^{n}Expri\;\times \;\beta i$$

On the other hand, the same formula and statistical methods were validated in the testing cohort. Based on the median risk score, both the training and validation cohorts were divided into low-risk and high-risk groups, and the survival difference between the low-risk and high-risk groups was compared via K–M analysis. The prognostic ability of the gene signature was further assessed using the ROC.

### 4.3. Correlation between the Gene Signature and UVM Common Mutations

In UVM, the treatment options and prognosis are closely related to the chromosomal aberrations and gene mutations. There were 60 patients with information on their chromosome 3 status in the testing cohort, and the information on chromosomes 3, 6p, and 8q was obtained from previous research [22,33]. The correlation between the gene signature and copy number aberrations was determined using the Pearson correlation coefficient in the training cohort. In addition, we utilized the “maftools” R package to visualize the mutation landscape and tumor mutation burden (TMB) of the CRTG signature in the TCGA-UVM cohort.

### 4.4. Gene Set Enrichment Analysis

To assess the possible mechanisms using the GSEA program (v3.0, DOI:10.1073/pnas.0506580102, http://software.broadinstitute.org/gsea/index.jsp, accessed on 31 July 2022), a GSEA was conducted between the low- and high-risk groups based on the HALLMARK gene set collection (v7.4, DOI:10.1093/bioinformatics/btr260, http://www.gsea-msigdb.org/gsea/downloads.jsp, accessed on 31 July 2022). The minimum number of genes was set at 5, the maximum at 5000, and the number of random sample permutations at 1000. Statistical significance was set at *p* < 0.05 and ab FDR of <0.25 was considered statistically significant.

### 4.5. Tumor Immune Microenvironment Analysis

The relative proportion of the 22 TICs was calculated using the CIBERSORT and ESTEMATE algorithms in the training cohort, and the relationship between the gene signature and the 22 TICs was assessed using the Spearman coefficient. We also compared the expression of the 35 checkpoint immunotherapy targets and 12 m6A-related genes between the high- and low-risk groups.

### 4.6. Cell Culture and siRNA Transfection

The uveal melanoma lineage cell line MUM-2B was obtained from the Hunan Clinical Research Center of Ophthalmic Disease. The MUM-2B cells were cultured in Roswell Park Memorial Institute (RPMI) 1640 (GIBCO, Grand Island, NY, USA) containing 10% fetal bovine serum (FBS), 100 U/mL penicillin, and 100 mg/mL streptomycin at 37 °C with 5% CO_2_. The MUM-2B cells were transfected with 50 nM small interfering (ACADSB siRNA, ORAI2 siRNA, SLC47A1 siRNA) and negative control RNA (Cohesion Biosciences, London, UK) using Lipofectamine^®^ RNAiMAX (Thermo Fisher Scientific, Waltham, MA, USA) for the knockdown experiments. After 24 h of transfection, the MUM-2B cells were collected for further experiments.

### 4.7. Western Blot

The Western blot analysis was performed as previously described (Huang et al., 2019). Briefly, 48 h after transfection, the proteins were extracted using radioimmunoprecipitation assay (RIPA) buffer (150 mM NaCl, 0.5% NP-40, 0.1% SDS, and 50 mM Tris–Cl, pH 7.5) and 1 mM PMSF, protease inhibitor, and phosphatase inhibitors. The protein concentrations were determined using a BCA Protein Quantitation Kit (Pierce, Thermo Fisher Scientific, MA, USA), and the protein lysates (40 μg/lane) were separated on 12% SDS-PAGE gels and transferred to PVDF membranes. The membranes were blocked with 5% non-fat milk in TBST (pH 7.4) at room temperature for 1 h and then incubated with the primary antibodies against the target proteins overnight at 4 °C. The membranes were washed thrice with TBST and incubated with the corresponding secondary antibodies conjugated to horseradish peroxidase for 1 h. In addition, the anti-GAPDH antibody and β-tubulin were used as the gel loading and transfer control, respectively. The band intensity was quantified using ImageJ2 software and normalized to that of the control group. The primary antibodies used were rabbit anti-SLC47A1, ACADSB, and ORAI2 (Signalway Antibody, Greenbelt, MD, USA).

### 4.8. Cell Proliferation

The proliferative capacity of the MUM-2B cells was assessed using a Cell Counting Kit-8 (CCK-8) assay. After 24 h of transfection, the cells were seeded in 96-well dishes at a density of 5 × 10^3^/well to continue to be cultured. After 0 h, 24 h, and 48 h, the MUM-2B cells were incubated with 10% CCK-8 solution for 1 h at 37 °C. The OD density at 450 nm was measured using a multifunctional microplate analyzer. The results are presented as the experimental group OD/negative control group OD × 100%.

### 4.9. Scratch Test

After 24 h of transfection, the MUM-2B cells were collected and implanted into a six-well plate and cultured to a 90–100% fusion degree. The bottom of each well was gently scratched with a 200 μL pipette tip. The scratch healing was examined under a microscope and photographed after a 24-h culture period. ImageJ2 software was used to measure the scratch width: mobility/% = (0 h scratch width − 24 h scratch width)/0 h scratch width × 100%.

### 4.10. Transwell Assay

After 24 h of transfection, the MUM-2B cells were collected and resuspended in serum-free medium. Next, 700 μL of medium containing 15% FBS was added to the lower chamber, and 200 μL of cell suspension (containing 1 × 10^5^ cells) was added to the upper chamber on top of a porous transwell membrane (8 μm pore size). The cells were incubated for 24 h. The cells were removed from the upper surface of the membrane. The migrated cells on the lower surface of the membrane were fixed and stained using crystal violet dye. The migrated cells were filmed under a microscope. ImageJ2 software was used to measure the cell area in each field.

### 4.11. The Effect of Cuproptosis on the Viability of MUM-2B Cells

The cuproptosis inducer (elesclomol–CuCl_2_) and its inhibitor (ammonium tetrathiomolybdate, TTM) were applied to test its effect on the MUM-2B cells. In short, 5 × 10^3^ cells were seeded on a 96-well plate and then cultured overnight in the medium containing 10% FBS. They were then treated with 5 μM TTM or 1 × PBS (vehicle). Pre-mixed elesclomol and CuCl_2_ with a 1:1 ratio (elesclomol–CuCl_2_) at a 200 nM concentration were added to each well, respectively, and pictures were taken at the 0 h, 8 h and 24 h time points under a microscope. The cell viability was assessed using a Cell Counting Kit-8 (CCK-8) assay at 24 h.

### 4.12. Statistical Analysis

The R package was used for all the statistical analyses. Here, *p* < 0.05, unless specifically indicated. The results are presented as the mean ± SD. The data between the two groups were compared using an independent sample *t*-test. The comparisons among multiple groups were assessed using a one-way analysis of variance (ANOVA), followed by Tukey’s post hoc test. The data at different time points were compared using a repeated-measures measures ANOVA, followed by a Bonferroni post hoc test.

## Figures and Tables

**Table 1:** Clinical characteristics of patients involved in the study.

Characteristic	Training Group (*n* = 80)	Testing Group (*n* = 63)	Total (*n* = 143)	*p* Value
Age				0.36
<65	36 (45.00%)	9 (11.25%)	45 (56.25%)	
≥65	24 (30.00%)	11 (13.75%)	35 (43.75%)	
Gender				1.00
Female	26 (32.50%)	9 (11.25%)	35 (43.75%)	
Male	34 (42.50%)	11 (13.75%)	45 (56.25%)	
T classification				0.58
T2	4 (5.00%)	1 (1.25%)	5 (6.25%)	
T3	25 (31.25%)	11 (13.75%)	36 (45.00%)	
T4	31 (38.75%)	8 (10.00%)	39 (48.75%)	
N classification				0.55
N0	56 (70.00%)	20 (25.00%)	76 (95.00%)	
NX	4 (5.00%)	0 (0.00 + 0%)	4 (5.00%)	
M classification				0.94
M0	55 (68.75%)	18 (22.50%)	73 (91.25%)	
M1	2 (2.50%)	1 (1.25%)	3 (3.75%)	
MX	3 (3.75%)	1 (1.25%)	4 (5.00%)	
Tumor stage				0.49
Stage II	27 (33.75%)	12 (15.00%)	39 (48.75%)	
Stage III	30 (37.50%)	7 (8.75%)	37 (46.25%)	
Stage IV	3 (3.75%)	1 (1.25%)	4 (5.00%)	
Tumor thickness, mm				0.69
<10	23 (28.75%)	6 (7.50%)	29 (36.25%)	
≥10	37 (46.25%)	14 (17.50%)	51 (63.75%)	
Tumor diameter, mm				0.16
<20	41 (51.25%)	18 (22.50%)	59 (73.75%)	
NA	1 (1.25%)	0 (0.00 + 0%)	1 (1.25%)	
≥20	18 (22.50%)	2 (2.50%)	20 (25.00%)	

NA: data not available.

## Data Availability

Publicly available datasets were analyzed in this study, which were downloaded from the TCGA (http://portal.gdc.cancer.gov/, TCGA-UVM, accessed on 31 July 2022) and the GEO (http://www.ncbi.nlm.nih.gov/geo/, GSE22138, accessed on 31 July 2022).

## Supplementary Materials

The following supporting information can be downloaded at https://www.mdpi.com/article/10.3390/ijms241411358/s1.
