# Supplementary material for: A Newly Established Cuproptosis-Related Gene Signature for Predicting Prognosis and Immune Infiltration in Uveal Melanoma
Source: Int J Mol Sci. 2023 Jul 12;24(14):11358. doi: 10.3390/ijms241411358 (PMC10379443; doi:10.3390/ijms241411358)

Table S1. The list of 80 prognostic CRGs

N0.	Gene Symbol	N0.	Gene Symbol	N0.	Gene Symbol	N0.	Gene Symbol
1	P2RX1	21	FUT4	41	SLC39A3	61	MCOLN1
2	NEU3	22	SLC47A1	42	AMY2B	62	CTNS
3	ATP6V0C	23	ADA	43	FBP1	63	DPAGT1
4	CNGA2	24	KCNA10	44	NAGPA	64	NUDT8
5	ATP1A4	25	NR1H2	45	ATP10B	65	PLA2G7
6	KCNJ1	26	KCNE3	46	MOCS1	66	ALG10B
7	ABHD5	27	ABCG2	47	GLB1L	67	AUH
8	STEAP4	28	IP6K3	48	PDE2A	68	SLC2A6
9	AGXT	29	SLC37A2	49	CSAD	69	KCNJ5
10	CHST10	30	HS3ST2	50	SLC39A11	70	NT5C1B
11	PLD1	31	ANKRD23	51	EPHX1	71	COQ9
12	PYCR1	32	KCNG1	52	PDXP	72	GCDH
13	SLC22A24	33	STARD5	53	EDN1	73	EHD1
14	LYZL6	34	SLC35E4	54	PIGC	74	SLC43A1
15	MIF	35	SULT1C3	55	DAGLA	75	CACNA2D2
16	TPH1	36	SLC7A9	56	SLC29A3	76	NDST1
17	CIDEA	37	TRPC1	57	ORAI2	77	DGKQ
18	GALNT14	38	SLC5A6	58	GLYCTK	78	STARD8
19	CHST13	39	ACADSB	59	SLC19A1	79	AADAT
20	SLC35C1	40	AQP11	60	PEMT	80	MTR

Table S2. GSEA analysis of UVM patients with high CRRS

NAME	NES	NOM p-val	FDR q-val
HALLMARK_OXIDATIVE_PHOSPHORYLATION	-2.3806	0.0000	0.0022
HALLMARK_IL6_JAK_STAT3_SIGNALING	-2.1914	0.0000	0.0098
HALLMARK_GLYCOLYSIS	-2.1549	0.0000	0.0107
HALLMARK_FATTY_ACID_METABOLISM	-2.1254	0.0000	0.0098
HALLMARK_MTORC1_SIGNALING	-2.1103	0.0020	0.0086
HALLMARK_PROTEIN_SECRETION	-2.0829	0.0021	0.0090
HALLMARK_IL2_STAT5_SIGNALING	-2.0406	0.0000	0.0121
HALLMARK_APOPTOSIS	-2.0293	0.0020	0.0116
HALLMARK_ADIPOGENESIS	-2.0097	0.0041	0.0120
HALLMARK_PEROXISOME	-1.9791	0.0000	0.0141
HALLMARK_REACTIVE_OXYGEN_SPECIES_PATHWAY	-1.9399	0.0000	0.0177
HALLMARK_PI3K_AKT_MTOR_SIGNALING	-1.9084	0.0021	0.0214
HALLMARK_INFLAMMATORY_RESPONSE	-1.9049	0.0210	0.0207
HALLMARK_COMPLEMENT	-1.8982	0.0153	0.0215
HALLMARK_ALLOGRAFT_REJECTION	-1.8542	0.0450	0.0273
HALLMARK_TNFA_SIGNALING_VIA_NFKB	-1.8253	0.0193	0.0316
HALLMARK_INTERFERON_GAMMA_RESPONSE	-1.8066	0.0464	0.0337
HALLMARK_UV_RESPONSE_UP	-1.7699	0.0061	0.0405
HALLMARK_KRAS_SIGNALING_UP	-1.7246	0.0270	0.0486
HALLMARK_ANGIOGENESIS	-1.6983	0.0431	0.0550
HALLMARK_UNFOLDED_PROTEIN_RESPONSE	-1.6927	0.0423	0.0541

HALLMARK_XENOBIOTIC_METABOLISM	-1.6746	0.0119	0.0572
HALLMARK_APICAL_SURFACE	-1.6110	0.0321	0.0770
HALLMARK_ESTROGEN_RESPONSE_LATE	-1.6065	0.0269	0.0754
HALLMARK_P53_PATHWAY	-1.5652	0.0317	0.0853

Figure S1. The biological effect of cuproptosis on MUM-2B cells. The cell viability was inhibited by cuproptosis.

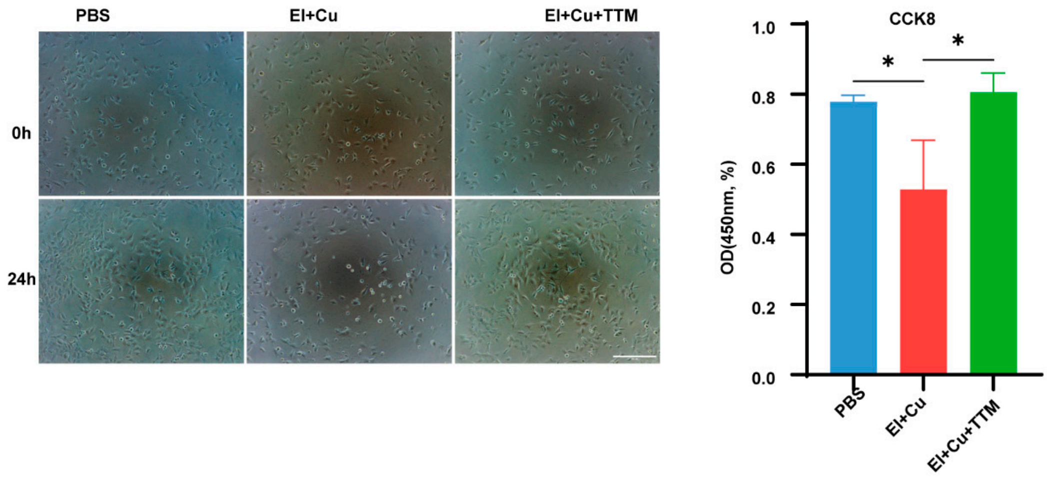

Supplement: Supplementary file 1 [file ijms-24-11358-s001.zip › ijms-2465550-supplementary.pdf]
